# EP17 Finding the road to sarcoidosis

**DOI:** 10.1093/rap/rkaa052.016

**Published:** 2020-11-03

**Authors:** Sharmin Nizam

**Affiliations:** Mid Yorkshire NHS, Wakefield, United Kingdom

## Abstract

**Case report - Introduction:**

Sarcoidosis is an autoimmune, multi-system condition in which the formation of non-caseating, granulomas is a key histological feature. Clinical presentation can be variable and may lead to a delay in recognition. Most cases will resolve with minimal or no intervention. Awareness of the condition and features helps guide long-term management and as illustrated in cases below, rheumatologists may often be involved in helping diagnose and coordinate the patient pathway.

**Case report - Case description:**

A 51- year old female ex-smoker experienced 6 months of fatigue, dry cough, mild exertional dyspnoea, sweats, mild weight loss and arthralgia after a cholecystectomy. She described lesions typical of erythema nodosum coinciding with a raised CRP (58g/L) which later normalised. Other than an elevated serum ACE (71 U/L), rest of tests were normal.

Plain chest radiograph was normal but a co-incidental CT abdomen for non-specific abdominal discomfort showed small volume abdominal lymphadenopathy. Further imaging showed bilateral mediastinal and hilar lymphadenopathy. Pulmonary function tests and joint ultrasound were normal. EBUS sampling (August 2014) excluded malignancy but confirmed sarcoid granulomas. She briefly required non-steroidals for arthralgia. Four years later, she is still well with resolution of lymphadenopathy.

A 41-year old male non-smoker presented with 6 weeks of bilateral heel pain followed by myalgia, weight loss, headaches, sweats, intermittent blurred vision, and a non-specific neck rash. He was afebrile with normal urinalysis, CRPs 24-39 mg/L, CCP, ANCA, ANA negative, (Serum ACE sample insufficient). Infection screening (including TB) was negative. Slit lamp examination was normal.

Trans-bronchial sampling of hilar lymphadenopathy seen on imaging excluded lymphoma but showed granulomas typical of sarcoidosis. The patient fully recovered within a few months without medication or recurrence.

A 63-year-old female was referred with ankle pain and swelling after 5 months of erythematous leg swelling treated initially as cellulitis. She also had bilateral, intermittent leg cramps and recent intermediate uveitis. She was positive for HLA B27 and ANA (homogenous speckled pattern) with a raised serum ACE (98 U). ANCA was negative, creatinine kinase normal. Background included treated squamous cell carcinoma and degenerative disc disease.

Ankle problems had resolved when seen possibly due to prednisolone for uveitis. EBUS sampling of bilateral hilar lymphadenopathy confirmed sarcoid histology. Since commencing azathioprine (50mg) for recurrent uveitis, she stays well.

**Case report - Discussion:**

Sarcoidosis is a granulomatous systemic disease thought to be Th-1 mediated but pathogenesis remains unclear. Heterogeneity in presentation and organ involvement may lead to delays or missed diagnoses. Like these cases, patients may have one or more presentations to various medical specialities before a link is made.

Careful note of antecedent history, current symptoms and examination findings can point towards a differential of sarcoid particularly if bilateral ankle involvement or typical skin lesions are present. Erythema nodosum can occur which the first case had described. Given the smoking and weight loss history, the differential of malignancy had to be excluded first. Sarcoid arthropathy, as seen in these cases, typically presents as arthralgia, myalgia, or arthritis in either acute or chronic form. Sometimes myopathy and bone involvement are seen though erosive disease is uncommon.

Cases often have minimal or no respiratory symptoms but chest imaging can pick up features including bilateral hilar lymphadenopathy (more than 75% of cases) and less commonly pulmonary parenchymal changes (nodules, ground glass changes, fibrosis) or pleural effusions.

Most cases will resolve over time with minimal intervention as in the first two cases. Some require non-steroidal anti-inflammatories. Steroids may be required if there are more inflammatory features affecting joints or other organs. Disease modifying therapies (biologic and non-biologic) have been used in more chronic or resistant cases.

Sarcoid may co-exist with or mimic other conditions. In the last case, the unifying diagnosis of uveitis, skin changes and joint involvement seems to be sarcoid. However, it was interesting that the patient had mixed serology and showed some features of a seronegative arthritis profile as spondyloarthritis and sacroiliitis have been reported with sarcoidosis.

Rheumatologists are often familiar with features of the condition. Thus, they can help link symptoms to guide appropriate investigations and further management with good outcomes.

**Case report - Key learning points**
 

- Sarcoidosis can have a heterogeneous presentation so may take a while for diagnosis to be made
- Respiratory symptoms may not be present despite findings on chest imaging
- Rheumatologists are often involved in diagnosis and treatment when patients with sarcoid related arthralgia or arthritis type symptoms get referred
- Most cases will resolve with minimal intervention
- Early recognition can streamline investigations and management subsequently improving the patient journey
- In cases with a mixed autoantibody profile, there may be a discussion on whether one or more conditions are present to explain all the features

